# Integrative modules for efficient genome engineering in yeast

**DOI:** 10.15698/mic2017.06.576

**Published:** 2017-06-05

**Authors:** Triana Amen, Daniel Kaganovich

**Affiliations:** 1 Department of Cell and Developmental Biology, Hebrew University of Jerusalem, Givat Ram, Jerusalem 91904, Israel.

**Keywords:** vector, bidirectional promoter, integrative plasmid, genetic integration, yeast, Saccharomyces cerevisiae

## Abstract

We present a set of vectors containing integrative modules for efficient genome integration into the commonly used selection marker loci of the yeast *Saccharomyces cerevisiae*. A fragment for genome integration is generated via PCR with a unique set of short primers and integrated into *HIS3*, *URA3*, *ADE2*, and *TRP1* loci. The desired level of expression can be achieved by using constitutive (*TEF1p*, *GPD1p*), inducible (*CUP1p*, *GAL1/10p*), and daughter-specific (*DSE4p*) promoters available in the modules. The reduced size of the integrative module compared to conventional integrative plasmids allows efficient integration of multiple fragments. We demonstrate the efficiency of this tool by simultaneously tagging markers of the nucleus, vacuole, actin, and peroxisomes with genomically integrated fluorophores. Improved integration of our new pDK plasmid series allows stable introduction of several genes and can be used for multi-color imaging. New bidirectional promoters (*TEF1p-GPD1p*, *TEF1p-CUP1p*, and *TEF1p-DSE4p*) allow tractable metabolic engineering.

## INTRODUCTION

*Saccharomyces cerevisiae* is an indispensable tool for high throughput studies of biological processes. The ever-increasing diversity of tools for yeast genome manipulation allows systematic examination of biological pathways in a highly physiological, cost-effective, and tractable manner 1,2,3,4,5,6,7. However, attempts to introduce multiple genes into chromosomal loci often encounter complications due to decreased efficiency and cloning limitations, such as overlaps in restriction site usage amongst multiple inserts 7,8. In order to overcome the difficulties of multiple gene integrations we sought to expand the variety of yeast integration cassettes with a specially designed pDK vector set.

Several methods enable rapid gene introduction. The most common ones are ectopic plasmid expression and chromosomal integration. Ectopic expression via multicopy or centromeric plasmids is often faster and easier than integration, but poses problems due to highly inhomogeneous expression 9,10. Hence, multi-color imaging and metabolic engineering in yeast often require stable integration of genes. Genome integration via homologous recombination has advantages over ectopic expression: e. g. stable strains, controlled copy number, and uniform expression 9.

**Figure 1:**
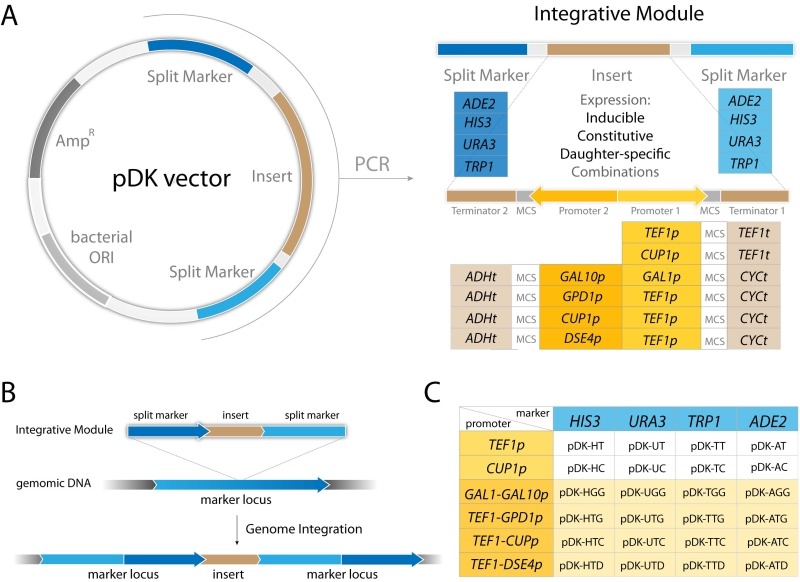
FIGURE 1: pDK vector series overview. **(A)** pDK vector with an integrative module flanked by a split marker. The customized insert is flanked by constitutive, inducible, or daughter-specific promoters. PCR is required for genetic integration. **(B)** Integration overview: the module is transformed into *S. cerevisiae* and inserted into the chromosome resulting in a marker locus duplication. **(C)** pDK vector set: constitutive, inducible, and four bi-directional promoter plasmids available for integration into four markers.

Several integration strategies are commonly employed. (1) Yeast integrative plasmids are introduced into the genome in linearized form 1. The linearized fragment contains the desired insert and selection marker, but also substantial superfluous genetic material including the bacterial selection marker and replication origins 1. These extraneous sequences may reduce integration efficiency 8. Additionally, the customized insert must not contain the restriction site used for plasmid linearization, adding further limitations. (2) Another frequently used strategy is PCR amplification using extended primers with homology regions allowing integration into any locus. Since the primers provide relatively short homology regions, the integration efficiency is comparatively low with this approach as well 8. Insertion into loci rather than selective markers does not pose a particular advantage since the selective marker locus still has to be present in the integrated fragment. (3) Recent advances in CRISPR/Cas9 genome engineering allow highly efficient introduction of multiple fragments with short homology regions and often without the need for a selective marker, however the strain must also carry Cas9 and gRNA expressing cassettes 4,11,12. (4) I-SceI-assisted integration has a significantly increased efficiency, but this approach also requires the additional expression of the meganuclease I-SceI locus 13. (5) Plas-mids carrying integrative cassettes excised by restriction with extended homology regions flanking the insert significantly improve integration efficiency 6,9,14. We were inspired by this last strategy and decided to create a set of yeast integration cassettes that contain extended homology regions corresponding to a common selective marker locus, which eliminates the need to have a marker locus inside the cassette. An advantage of not having a selective marker locus inside the cassette reduces its size and potentially increases integration efficiency 8. To reduce multiple integration time and to maximize available markers we constructed novel bidirectional promoter sets (Figure 1A).

The pDK series includes 24 plasmids which carry an integrative module for 4 common genetic markers (*HIS3*, *URA3*, *ADE2*, and *TRP1*). Constitutive (*TEF1p*, *GPD1p*), daughter specific (*DSE4p*) and inducible (*CUP1p* and bidirectional *GAL1/10p*) promoters flank multiple cloning sites. We also include 4 bidirectional promoters (*GAL1p-GAL10p*, *TEF1p-GPD1p*, *TEF1p-CUP1p*, and *TEF1p-DSE4p*) which allow multiple integrations (Figure 1, Table 1). PCR is carried out with short primers specific to a selectable marker in order to integrate the module (see Supplemental Table 1). The pDK set can be used for multi-color imaging, metabolic engineering, or any set of experiments that require stable genomic integration of one or more genes.

**Table 1:** pDK plasmid series with integrative modules.

			Locus	
**Module**	***HIS3***	***URA3***	***TRP1***	***ADE2***
*TEF1p*-MCS-*TEF1t*	pDK-HT	pDK-UT	pDK-TT	pDK-AT
*CUP1p*-MCS-*TEF1t*	pDK-HC	pDK-UC	pDK-TC	pDK-AC
*GAL1p*-MCS(1)-*CYC1t* *GAL10p*-MCS(2)-*ADHt*	pDK-HGG	pDK-UG	pDK-TG	pDK-AG
*TEF1p*-MCS(1)-*CYC1t* *GPD1p*-MCS(2)-*ADHt*	pDK-HTG	pDK-UTG	pDK-TTG	pDK-ATG
*TEF1p* -MCS(1)-*CYC1t* *CUP1p*-MCS(2)-*ADHt*	pDK-HTC	pDK-UTC	pDK-TTC	pDK-ATC
*TEF1p* -MCS(1)-*CYC1t* *DSE4p*-MCS(2)-*ADHt*	pDK-HTD	pDK-UTD	pDK-TTD	pDK-ATD

## RESULTS AND DISCUSSION

### Vector overview

We designed 24 pDK plasmids for stable expression of multiple genes in the yeast *S. cerevisiae* (Figure 1, Table 1). Vectors can be used for stable integration into 4 common *S. cerevisiae* selective marker loci *HIS3*, *URA3*, *ADE2*, and* TRP1*. Plasmid variations include 6 promoters (Figure 1C): Constitutive *TEF1p*, inducible *CUP1p*, inducible bidirectional *GAL1-10p* in opposing orientation, constitutive bidirectional *TEF1-GPD1p*, constitutive-inducible bidirectional *TEF1-CUP1p*, constitutive-daughter-specific bidirectional *TEF1-DSE4p* (bidirectional promoter sets have two multiple cloning sites (MCS) in opposing orientation). The markers are split into two parts (see plasmid construction for details) and are flanking the region containing a promoter, MCS, and a terminator (Figure 1A). The fragment is amplified with primers specific to the marker (Supplemental Table 1). The orientation of the split markers allows integration of amplified region in a traditional homologous recombination driven way, resulting in doubling of the marker region (Figure 1B).

### Integrative modules allow stable and efficient integration of multiple inserts

pDK vectors were examined for integration efficiency, correct integration, and stability of the insert. 24 integrative modules of pDK series were transformed into the W303 strain. pDK integrative modules have comparable integration efficiencies (Supplemental Figure 1A). Although double integration is possible we recommend sequential integration and using bidirectional promoters to expedite the work flow. We also compared the integration efficiency to other integration strategies: (1) pRS series - conventional linearized integrative plasmids 1, (2) EasyClone strategy - extended homology regions, excisable marker and restriction based integration 6, and (3) extended primers - 45bp homology region. In general, extended homology region based strategies provide higher efficiency of integration. pDK series integration is more effective than pRS series, and is comparable with EasyClone efficiency, which also has a reduced insert size but relies on restriction based integration.

To evaluate integration stability, a GFP reporter was cloned into the pDK series. The stability of the integration was tested by avoiding selection for more than 80 generations and then counting fluorescent cells (Supplemental Figure 1B). Modules are integrated with 95 - 100% accuracy into the marker region as verified by PCR (Supplemental Figure 1B). Marker based integration is advantageous because it provides accurate targeting compared to the CRISPR/Cas9 strategy 15. We also tested the stability of multiple integrations, since many of them carry same promoters and/or terminators, which can potentially lead to a loop-out of the fragments. Single and multiple integrations provide reliable homogenous expression levels under non-selective conditions (Supplemental Figure 1B, C).

### Application of pDK plasmids to multi-color imaging reveals order of organelle inheritance

**Figure 2:**
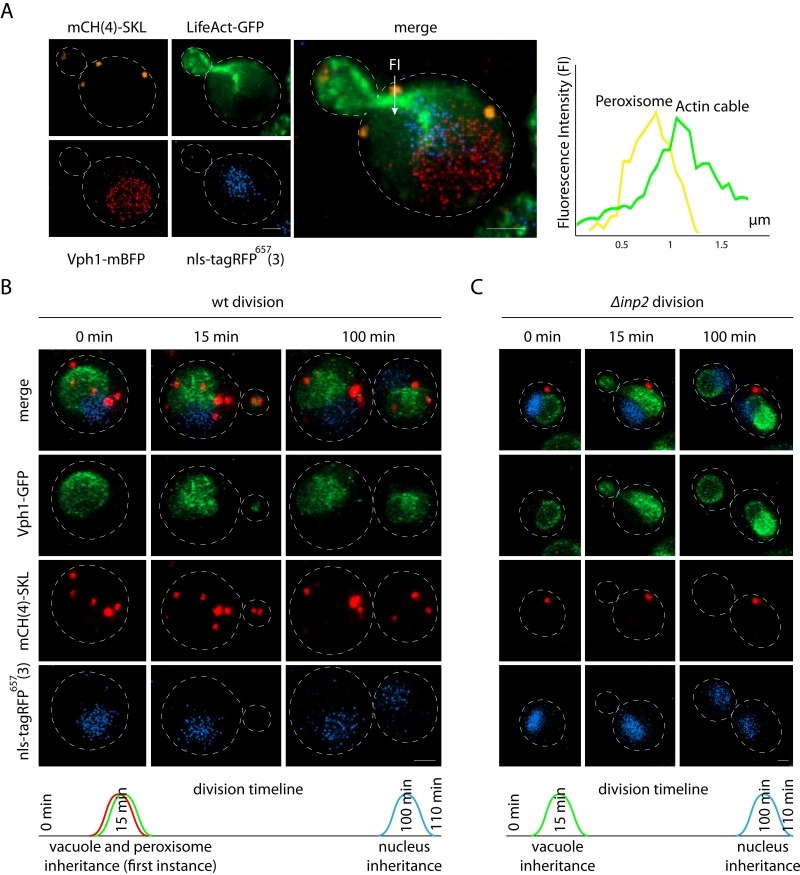
FIGURE 2: Proof of concept - Organelle inheritance order during division. **(A)** *In vivo* 4-color imaging of peroxisome (SKL signal C-terminally fused to 4 mCherry (mCH) on pDK-UT), actin (LifeAct fused to GFP on pDK-AT), nucleus (SV40 nls signal fused to 3 far-red fluorophores on pDK-HT), and vacuole (*VPH1* endogenously tagged with mBFP), the scale bar is 1 µm. The graph represents proximity of peroxisome to actin cable during division, Fluorescence Intensity (FI) was calculated along the x axes (arrow on the merge image) for red (peroxisome) and green (actin) channels. **(B)** The timeline of organelle inheritance in the wild type (wt) strain, scale bar is 1 µm, time points represent the average (n = 30) time of organelle inheritance from the start of division, strain carries SKL signal C-terminally fused to 4 mCherry (mCH) on pDK-UT module, SV40 nls signal fused to 3 far-red fluorophores on pDK-HT module, and *VPH1* endogenously tagged with GFP. **(C) **The timeline of the organelle inheritance in the Δ*inp2* strain, scale bar is 1 µm.

As proof of concept for multi-color imaging we constructed peroxisomal, nuclear, and actin 16 markers using the pDK vector series and used the strain for 3D time-lapse microscopy (4D imaging) (Figure 2) 17. Nuclear localization signal fused to a far-red fluorophore was used to visualize the nucleus. Peroxisome localization signal - tripeptide SKL was fused to the mCherry C-terminus to visualize peroxisomes. We also used LifeAct fused to GFP to visualize actin in living yeast 16. The vacuolar marker *VPH1* was endogenously tagged with mBFP or GFP. Peroxisomes are known to use actin cables for inheritance 18. Inp2, an integral peroxisomal membrane protein, binds the Myo2 motor ensuring localization to the bud 19,20. Most of the organelles in yeast use actin cables for bud trafficking during division 21. To determine whether organelle inheritance proceeds in a parallel or a serial way we examined the inheritance of the vacuole, peroxisomes, and the nucleus (Figure 2, Supplemental movie “wtdivision”). The nucleus itself is inherited in a microtubule dependent manner, however the astral microtubules are delivered on actin to the bud 22. Multi-color imaging revealed that the first instance of peroxisome inheritance happens in parallel with the vacuole in the first 15 min of cell division (Figure 2B). Deletion of *INP2* abolishes peroxisome inheritance, which has no effect on the time of vacuole inheritance 19 (Figure 2C, Supplemental movie “inpdivision”). Interestingly, the nucleus is inherited prior to the end of cell division. The integrative vector series that we have developed allows for efficient integration of up to 8 markers, enabling us to image cellular compartments simultaneously.

### Application of pDK plasmids to multi-copy integration

**Figure 3:**
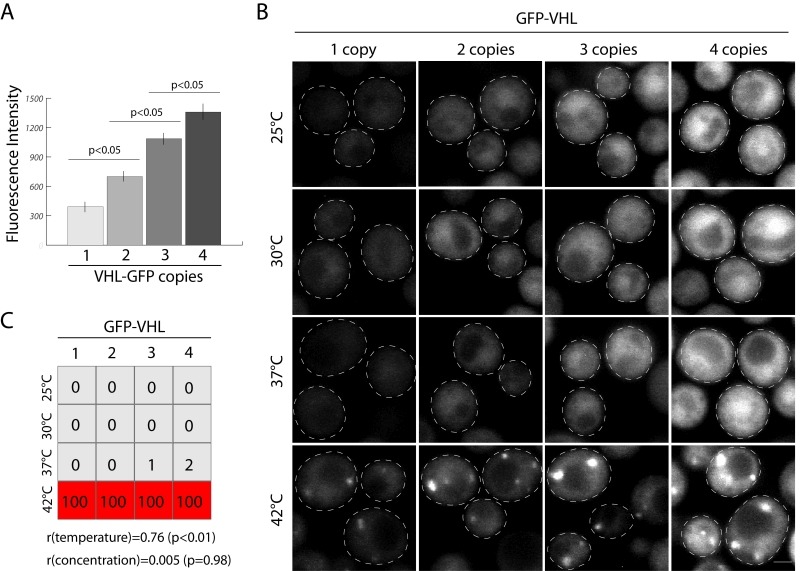
FIGURE 3: Proof of concept - Concentration does not correlate with inclusion formation. **(A)** Gradual increase in the amount of GFP-VHL according to integrated copies, Fluorescence Intensity was quantified in 30 single cells in the population, the average and standard errors are represented on the graph. **(B)** Confocal images of cells carrying 4 copies of GFP-VHL on pDK-AC, TC, UC, HC modules subjected to the range of temperatures for 1h, scale bar 1 µm. **(C)** Quantification of inclusion forming cells in the population according to the change in the temperature and the concentration, the numbers represent % of the cells with inclusions (n = 300), correlation coefficient of aggregation and temperature (r(temperature)) and aggregation and concentration (r(concentration)) is provided under the diagram.

As proof of concept of exquisitely controlled inducible expression of a gene using multiple integrations we inserted GFP tagged VHL under inducible *CUP1* promoter in 4 marker loci. The von Hippel Landau tumor suppressor (VHL) has often been used as a model substrate to study protein aggregation in yeast 23. Carefully controlling expression levels of integrated proteins is essential to many studies. In the case of VHL and similar proteins, increased protein concentration results in gradual protein self-association with subsequent decrease in the monomer fraction *in vitro*. This leads to the formation of protein inclusions or aggregates. To test if there is a correlation of misfolded protein concentration and inclusion formation *in vivo* we introduced 1-4 copies of GFP-VHL under *CUP1p* (Figure 3A, B) in yeast. We scored inclusion formation as a function of concentration and temperature. Aggregation strongly correlates with an increase in temperature, r(temperature) = 0.76 (p < 0.05), but has no significant correlation with VHL concentration, r(concentration) = 0.005 (p = 0.98). This could indicate a number of things. One interpretation of these data is that, regardless of the concentration, GFP-VHL forms a small percentage of overall protein content in inclusions (because of the abundance of misfolded and unstructured proteins in yeast). Another possibility is that without significant heat shock the quality control system is able to degrade a spectrum of misfolded VHL concentrations, but at higher temperatures it becomes uniformly inhibited. Additional experiments could make similar titrations of proteasome and chaperone activity. Together, these data are compelling as proof-of-concept for multi-gene integrations.

### Bidirectional promoter sets

**Figure 4:**
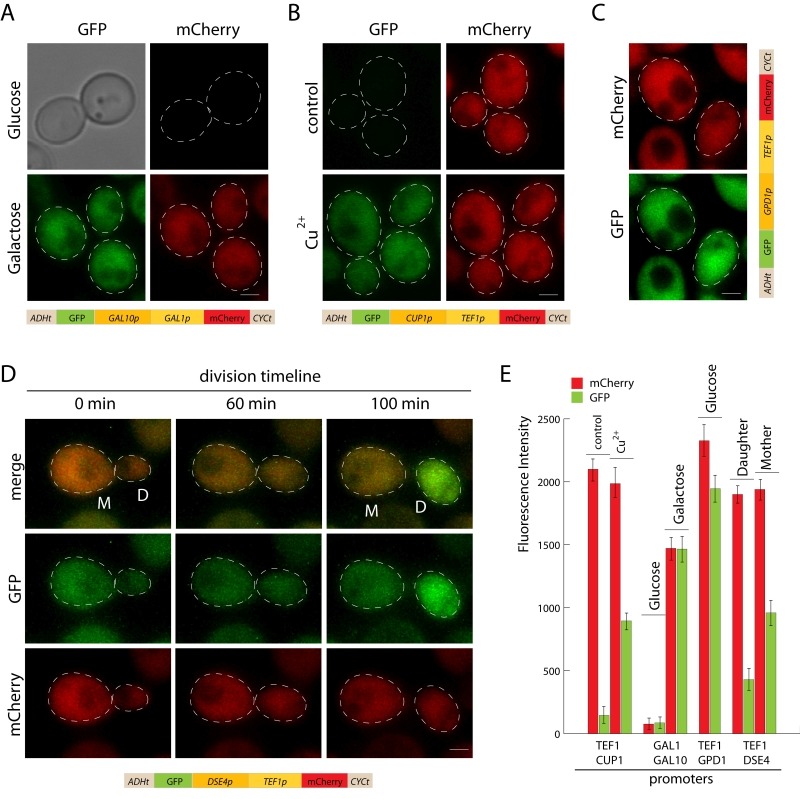
FIGURE 4: Bidirectional promoter reporters. **(A) **Galactose inducible bidirectional promoter, cells carrying pDK-HGG-GFP-mCherry were induced with galactose or grown on glucose for 6 hours, scale bar 1 µm. **(B) **Constitutive-inducible promoter, cells carrying pDK-HTC-GFP-mCherry were grown with and without (control) copper2+ for 4 hours, scale bar 1 µm. **(C)** Constitutive bidirectional promoter, cells carrying pDK-HTG-GFP-mCherry were grown on glucose containing medium to middle log phase, scale bar 1 µm. **(D)** Daughter-specific-constitutive bidirectional promoter, cells carrying pDK-HTD-GFP-mCherry module were grown on glucose containing medium on the microscope, frames are shown (0 min, 60 min, 100 min). **(E)** Comparison of fluorescence intensity of bidirectional reporters, the graph shows the average of fluorescence intensity of 20 cells and standard error.

To improve the time of strain construction, we built versatile bidirectional promoter sets, allowing integration of two inserts under different promoters at the same time. The promoters are positioned in the opposite orientation (Figure 1A). Previously described *GAL1p/GAL10p* and 3 novel promoters: *TEF1p-GPD1p*, *TEF1p-CUP1p*, and *TEF1p-DSE4p* were introduced into pDK series. To validate promoter sets we constructed GFP/mCherry reporters and visualized the cells during the log phase (Figure 4). Bidirectional promoters allow controlled inducible, semi-inducible, constitutive and daughter-specific expression (Figure 4A-D). Not only do they facilitate strain construction, bidirectional sets can also be used for cell sorting (daughter/mother cells), studying aging, or screening yeast genome collections 24,25.

In summary, the pDK vector series allows for efficient multiple integrations and thus is a useful tool for multi-color imaging, metabolic engineering, controlled expression of genes of interest, and stable yeast strain production. We therefore hope that pDK vectors will be a useful tool for the yeast community.

## MATERIALS AND METHODS

### Strains and media

We used standard conditions for culturing yeast and bacterial cells 26. Yeast were grown in the selective medium (1.7 g/l yeast nitrogen base without amino acids and ammonium sulfate (Difco Laboratories), 5 g/l ammonium sulphate, 0.77 g/l complete, 2 g/l amino acids supplement powder mix 27, 20 g/l glucose, and 20 g/l agar for the solid medium) or rich medium (20 g/l Peptone, 10 g/l yeast extract, 20 g/l glucose, and 20 g/l agar for the solid medium). Galactose induction was performed on selective medium supplemented with 20 g/l galactose instead of glucose for 6 hours. *CUP1p* induction was performed by addition of 50 µM Cupric Sulphate to the selective medium and growing cells for 4 hours. Plasmids were constructed using *Escherichia coli* strain DH5α. Yeast strain W303-1B (*MATα leu2-3,112 trp1-1 can1-100 ura3-1 ade2-1 his3-11,15*) was used for the experiments.

**Table 2:** Strains used in this study.

**Strain**	**Genotype**
BY4741	*MATa his3*Δ*0 leu2*Δ*0 met15*Δ*0 ura3*Δ*0*
W303α	*MATα leu2-3,112 trp1-1 can1-100 ura3-1 ade2-1 his3-11,15*
yDK - *inp2*, nuc, pex, vac	W303α *inp2*Δ*::KanMX his3-11,15::nls-(tagRFP657)_3_::HIS3 ura3-1:(mCherry)_3_-SKL::URA3 VPH1-GFP-HPH*
yDK - nuc, pex, vac	W303α *his3-11,15::nls-(tafRFP657)_3_::HIS3 ura3-1:(mCherry)_3_-SKL::URA3 VPH1-GFP-HPH*
yDK - nuc, pex, la, vac	W303α *his3-11,15::nls-(tafRFP657)_3_::HIS3 ura3-1:(mCherry)_3_-SKL::URA3 ade2-1::LifeAct-GFP::ADE2 VPH1-mBFP-HPH*
yDK - HHCTGV	W303α *his3-11,15::CUP1p-GFP-VHL-TEF1t::HIS3*
yDK – HHCTGV, UUCTGV	W303α *his3-11,15::CUP1p-GFP-VHL-TEF1t::HIS3 ura3-1::CUP1p-GFP-VHL-TEF1t::URA3*
yDK – HHCTGV, UUCTGV, TTCTGV	W303α *his3-11,15::CUP1p-GFP-VHL-TEF1t::HIS3 ura3-1::CUP1p-GFP-VHL-TEF1t::URA3 trp1-1::CUP1p-GFP-VHL-TEF1t::TRP1*
yDK – HHCTGV, UUCTGV, TTCTGV, AACTGV	W303α *his3-11,15::CUP1p-GFP-VHL-TEF1t::HIS3 ura3-1::CUP1p-GFP-VHL-TEF1t::URA3 trp1-1::CUP1p-GFP-VHL-TEF1t::TRP1 ade2-1::CUP1p-GFP-VHL-TEF1t::ADE2*

### Plasmid construction

pUC19 plasmid 28 was used as a backbone for pDK vectors. Primers used in this study are summarized in supplemental tables: 1 - primers used for yeast integration, 2 - primers used for construction of plasmids. Markers loci (including promoters and terminators) were split in the following parts: *HIS3* (after 286bp of the ORF), *URA3* (after 420 bp of the ORF), *TRP1* (after 224 bp of the ORF), *ADE2* (after 462 bp of the ORF). Split fragments were amplified with: HIS3F1/R1 and HIS3F2/R2, URA3F1/R1 and URA3F2/R2, TRP1F1/R1 and TRP1F2/R2, ADE2F1/R1 and ADE2F2/R2, and cloned into pUC19’s *Pci*I/*Hin*dIII and *Pfo*I/*Eco*RI, *Eco*RI/*Pfo*I and *Pci*I/*Hin*dIII, *Hin*dIII/*Pci*I and *Pfo*I/*Eco*RI, *Hin*dIII/*Pci*I and *Pfo*I/*Eco*RI respectively, resulting in the plasmids pDK-HH, pDK-UU, pDK-TT, and pDK-AA. Promoters and terminators (*TEF1p*, *CUP1p*, *TEF1t*) were amplified with: TEF1PF/R, CUP1PF/R, TEF1TF/R primers and cloned into pUC19’s *Nde*I/*Eco*RI, *Nde*I/*Eco*RI, and *Sal*I/*Hin*dIII sites, respectively, resulting in pUC19-*TEF1p*-*TEF1t* and pUC19-*CUP1p*-*TEF1t* plasmids. *TEF1p*-*TEF1t*, *CUP1p*-*TEF1t* and *GAL1*/*10p*-*ADH1*/*CYC1t* modules were amplified from previously constructed pUC19-*TEF1p*-*TEF1t* and pUC19-*CUP1p*-*TEF1t*, and pESC-URA (Agilent) vectors using the primers: CUPTEFHHF, CUPTEFUUF, CUPTEFTTF, CUPTEFAAF and CUPTEFR for *TEF1p*-*TEF1 *and, *CUP1p*-*TEF1t* modules, and GALHHF, GALUUF, GALTTF, GALAAF and GALR for *GAL1/10p*-*ADH1/CYC1t* module, and cloned using Gibson Assembly mix (NEB) into pDK-HH, pDK-UU, pDK-TT, and pDK-AA vectors linearized with *Eco*RI/*Sal*I restriction enzymes resulting in 12 pDK plasmids. Plasmid names contain the first letter of the marker followed by the first letter/s of the promoter/s (Table 1). *TEF1* promoter was amplified with TEFbF/R primers and cloned into pESC-URA *Age*I/*Bam*HI sites resulting in the pESC-TEFp plasmid. *CUP1*, *DSE4*, and *GPD1* promoters were amplified with CUPbF/R, DSEbF/R, and GPDbF/R primers and cloned into the pESC-TEFp vector into *Age*I/*Eco*RI sites resulting in pESC-TG, TC and TD plasmids carrying bidirectional promoters. Bidirectional promoters were subcloned using *Bam*HI/*Eco*RI into pDK-HG/UG/TG/AG, resulting in 12 bidirectional promoter sets (Table 1).

The nuclear cellular marker plasmid was constructed by cloning the SV40 nuclear localization signal fused to (tagRFP657)4 29 into pDK-HT vector *Eco*RI/*Sma*I sites. The peroxisome marker was constructed by cloning the (mCherry)4-SKL sequence into pDK-UT plasmid. Actin was visualized via the LifeAct fragment fused to GFP 16, LifeAct-GFP was cloned into the pDK-AT vector. VHL plasmids were constructed by cloning the GFP-VHL sequence into pDK-HC, pDK-UC, pDK-AC, and pDK-TC vectors. pCfB2513 was a gift from Irina Borodina (Addgene plasmid # 67543).

For endogenous tagging we modified the pKT127 30 plasmid by inserting the mBFP sequence or GFP sequence into *Pac*I/*Asc*I sites and *HPH* marker sequence into *Bgl*II/*Pme*I sites. GFP reporters were constructing by cloning GFP into *Sac*I/*Xma*I sites of pDK-HT/HC, and *Bam*HI/*Xma*I sites of pDK-HGG with primers GFPxR, GFPbF, GFPsF and subcloning the fragment containing the marker across different marker plasmids and bidirectional promoter ones. mCherry was amplified with primers CHeF and CHnR and cloned into the *Eco*I/*Spe*I sites of pDK-HGG-GFP, and subcloned to pDK-HTC-GFP, pDK-HTG-GFP, and pDK-HTD-GFP.

### Strain construction

We introduced the *INP2* deletion using a PCR based deletion strategy 31 and primers delINPF/R. Gene deletions were verified by PCR. Strains with integrative modules were constructed by transforming yeast with a PCR fragment obtained from a corresponding plasmid with a set of primers listed in Table 1. For comparison experiments pRS plasmids were linearized in the marker locus prior to integration, pRS303 and pRS306 with *Pst*I restriction enzyme, and pRS304 with *Pml*I enzyme.

VPH1 was tagged using modified pKT127 plasmid and eVPHF/R primers.

### Protocol for yeast integration

The fragment for genomic integration is generated via PCR with primers listed in Table 2 using the following parameters (95°C- 5’, [B95°C- 30’’, 62°C- 30’’ (increment 0.8°C per cycle), 72°C- X min (X = length of the fragment in kb)">95°C- 30’’, 62°C- 30’’ (increment 0.8°C per cycle), 72°C- X min (X = length of the fragment in kb)] 25 cycles, 72°C- 5’), and high fidelity polymerase generating blunt-end products, e.g. KAPA HiFi DNA Polymerase (KAPA Biosystems). A PCR protocol with fixed primer binding time can also be used. Up to 2 μg of PCR product is transformed using LiAc/PEG transformation 32 with modifications - 50 μl of DMSO is added prior to heat shock, heat shock time is reduced to 15 min at 42°C. 20 random clones carrying integrative modules were verified by PCR.

### Stability of integration

Yeast strains were grown in rich medium in three replicates. The culture was diluted 1/100 every 24 hours for 10 days. The culture was analyzed by reporter fluorescence on the first and tenth day.

### Confocal Microscopy

For imaging yeast cells were grown to mid-log phase and seeded on concanavalin A (Sigma) coated 4-well microscope plates (IBIDI). For induction galactose rich media (division experiments, Figure 2) or minimal media with 50 µm Cu^2+^ (Figure 3) were used. Copper induced cells were grown in 25°C to mid-log phase and then incubated for 1h at indicated temperatures (30, 37, 42°C). Confocal 3D images and movies were acquired using a dual point-scanning Nikon A1R-si microscope equipped with a PInano Piezo stage (MCL), using a 60 x PlanApo VC oil objective NA 1.40. Movies were acquired in resonant-scanning mode. Image processing was performed using NIS-Elements software.

### Statistics

The experiments were repeated at least 3 times. Multiple correlation coefficients were calculated for 3 variables (aggregation, temperature, concentration), with subsequent regression analysis to determine p-values. Standard comparisons were performed using t-test.

## SUPPLEMENTAL MATERIAL

Click here for supplemental data file.

Click here for supplemental movie „wtdivision“.

Click here for supplemental movie „inpdivision“.

All supplemental data for this article are also available online at http://microbialcell.com/researcharticles/integrative-modules-for-efficient-genome-engineering-in-yeast/.
