# Supplementary material for: Integrative modules for efficient genome engineering in yeast
Source: Microb Cell. 2017 Jun 5;4(6):182–90. doi: 10.15698/mic2017.06.576 (PMC5473690; doi:10.15698/mic2017.06.576)
Supplement: Supplementary file 1 [file mic-04-182-s01.pdf]

A

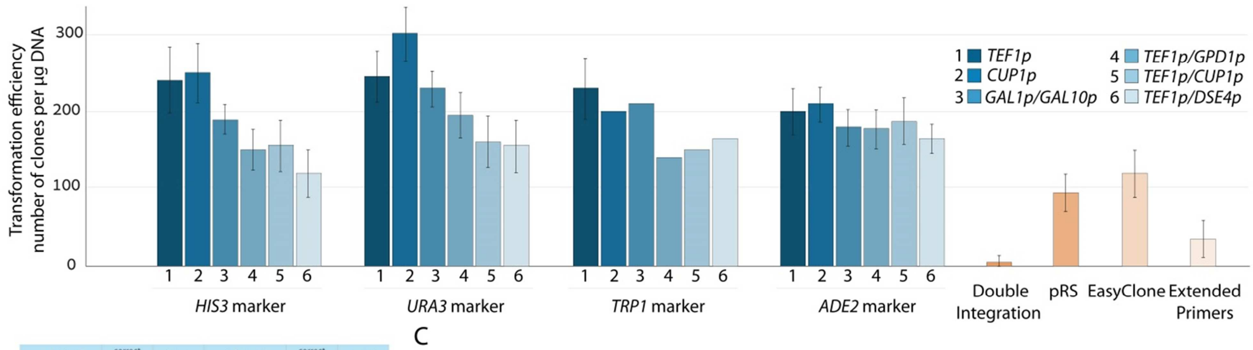

B

plasmid	correct integration	stability	plasmid	correct integration	stability
pDK-HT	100	100	pDK-TT	100	100
pDK-HC	100	100	pDK-TC	100	100
pDK-HGG	100	100	pDK-TGG	100	100
pDK-HTG	100	100	pDK-TTG	100	100
pDK-HTC	100	100	pDK-TTC	100	100
pDK-HTD	100	100	pDK-TTD	100	100
pDK-UT	100	100	pDK-AT	95	100
pDK-UC	100	100	pDK-AC	95	100
pDK-UGG	100	100	pDK-AGG	100	100
pDK-UTG	100	100	pDK-ATG	95	100
pDK-UTC	100	100	pDK-ATC	95	100
pDK-UTD	100	100	pDK-ATD	100	100

C

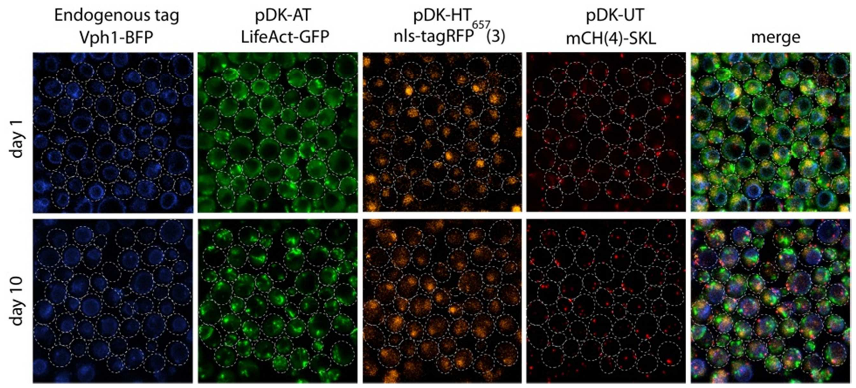

Supplemental FIGURE 1: Testing the pDK vector series. (A) Comparison of integration efficiency of pDK plasmids (24), double integration (using pDK-HT and pDK-UT), EasyClone plasmid pCfB2513, pRS series (pRS303, pRS304, pRS306), and extended primers (integration of BFP-HPH fragment with 45bp overhangs, see methods for details). Transformation efficiency is expressed as a number of transformants per µg DNA, for $5*10^7$ cells per transformation, error bars represent standard deviation. **(B)** Fidelity of integration was evaluated by PCR from 20 clones, and stability of integration was evaluated by fluorescence intensity of GFP reporter after 10 days of growing cells in non-selective rich medium. **(C)** Stability of multiple plasmids carrying the same promoter and terminator was evaluated by GFP fluorescence after 10 days of growing in non-selective medium.

Supplemental TABLE 1. Oligonucleotides used for generating the fragment for integration.

	Primers for integration	
**Locus**	**Forward primer sequence**	**Reverse primer sequence**
**HIS3** (for pDK-HT, pDK-HC, pDK-HGG, pDK-HTG, pDK-HTC, pDK-HTD)	5'-gcgggattgctctcg-3'	5'-agtcttcagtggtgtgatg-3'
**URA3** (for pDK-UT, pDK-UC, pDK-UGG, pDK-UTG, pDK-UTC, pDK-UTD)	5'-tacttcttctgccgcc-3'	5'-acaaaggaacctagaggc-3'
**TRP1** (for pDK-TT, pDK-TC, pDK-TGG, pDK-TTG, pDK-TTC, pDK-TTD)	5'-cgtgtttcgtaatcaacc-3'	5'-ccaaccaagtatttcgg-3'
**ADE2** (for pDK-AT, pDK-AC, pDK-AGG, pDK-ATG, pDK-ATC, pdK-ATD))	5'-gatggaagaggtaacttcg-3'	5'-gtatgccaaagtcctcg-3'

Supplemental TABLE 2. Oligonucleotides used for plasmid construction.

Primer	Sequence	Primer	Sequence
**HIS3F1**	5'-gactaagcttcgttttaagagcttggtg-3'	**GALAAF**	5'-gctcaatgcagaagaattgctatacctgagaaagcaac-3'
**HIS3R1**	5'-gactacatgtagtcttcagtggtgtg-3'	**GALTTF**	5'-ctatgcggtgtgagaattgctatacctgagaaagcaac-3'
**HIS3F2**	5'-gacttcccggagcgggattgctctc-3'	**CUPTEFR**	5'-catgcctgcaggtcgacagtatagcgaccagc-3'
**HIS3R2**	5'-gactgaattcccgtcgagttcaagag-3'	**CUPTEFHHF**	5'-gaactcgacgggaattgattgtactgagagtgcac-3'
**URA3F1**	5'-gatcgaattcccataccacagcttttc-3'	**CUPTEFUUF**	5'-gctgtggtatgggaattgattgtactgagagtgcac-3'
**URA3R1**	5'-gatctcccggatacttcttctgccgc-3'	**CUPTEFAAF**	5'-ggcggctcaatgcagaagaattgattgtactgagagtg-3'
**URA3F2**	5'-gatcacatgtacaaaggaacctagagg-3'	**CUPTEFTTF**	5'-ctatgcggtgtgagaattattgtactgagagtgcac-3'
**URA3R2**	5'-gatcaagctttcacaccgcatagggg-3'	**CUPbF**	5'-gatcaccggtggctgatatcttagcctt-3'
**TRP1F1**	5'-gatcaagcttgagagtgcaccataaacg-3'	**CUPbR**	5'-gatcgaattcgatgacttctatatgatattgc-3'
**TRP1R1**	5'-gatcacatgtccaaccaagtatttcgg-3'	**GPDbF**	5'-gatcaccggttcattatcaatactgccatttc-3'
**TRP1F2**	5'-tcccggacgtgtttcgtaatcaacc-3'	**GPDbR**	5'-gatcgaattctttgtttgtttatgtgtgttt-3'
**TRP1R2**	5'-gatcgaattctcacaccgcataggc-3'	**DSEbF**	5'-gatcaccggtaaaaaacctccccatcaaa-3'
**ADE2F1**	5'-gatcaagcttcttttgatgcggaattgac-3'	**DSEbR**	5'-gatcgaattcagctgtttttttgttttct-3'
**ADE2R1**	5'-gatcacatgtgtatgccaaagtcctcg-3'	**TEFbF**	5'-gatcaccggtgacatggaggcccag-3'
**ADE2F2**	5'-gatctcccggagatggaagaggtaacttcg-3'	**TEFbR**	5'-gatcggatccggttgtttatgttcggatg-3'
**ADE2R2**	5'-gatcgaattcttctgcattgagccgc-3'	**GFPxR**	5'- gatccccgggttacttgtacagctcgtcc-3'
**TEF1PF**	5'-gatcaccggtcatatggacatggaggcccag-3'	**GFPbF**	5'- gatcGGATCCatggtgagcaagggcgag-3'
**TEF1PR**	5'-gatcgaattcggttgtttatgttcggatg-3'	**GFPsF**	5'-gactGAGCTCatggtgagcaagggcgag-3'
**CUP1PF**	5'-gatccatatgggctgatatcttagccttg-3'	**CHeF**	5'-gatcggatccgtgagcaagggcgag-3'
**CUP1PF**	5'-gatcgaattcgattgattgattgtacag-3'	**CHnR**	5'-gatcgctagcttacttgtacagctcgtcc-3'
**TEF1TF**	5'-gatcgtcgacactgacaataaaaagattc-3'	**delINPF**	5-gtttgtttttacttacttgtgaaacgtttgttgattaacttaatacgctagggtaacaggg-3'
**TEF1TR**	5'-gatcaagcttcagtatagcgaccag-3'	**delINPR**	5'-ttaaagtgtaattagttatttcaaagtacatattaaaatatattagatgaattcgagctcg-3'
**GALR**	5'-atgcctgcaggtcgacttcgagcgtcccaa-3'	**eVPHF**	5'-gacatggaagtcgctgttgctagtgcaagctcttccgcttcagcggtg acggtgctggttta-3'
**GALHHF**	5'-gaactcgacgggaattgctatacctgagaaagc-3'	**eVPHR**	5'-aatgaagtacttaaatgtttcgctttttttaaaagtcctcaaaattcga tgaattcgagctcg-3'
**GALUUF**	5'-ctgtggtatgggaattgctatacctgagaaagcaac-3'		
